# Is an additional cannulated screw necessary for unstable femoral neck fractures with comminuted posteromedial cortex by femoral neck system (FNS) fixation? a biomechanical and clinical study

**DOI:** 10.3389/fbioe.2025.1658728

**Published:** 2025-11-14

**Authors:** Jixing Fan, Youliang Hao, Yuan Cao, Zengzhen Cui, Yang Lv, Fang Zhou

**Affiliations:** 1 Department of Orthopedics, Peking University Third Hospital, Beijing, China; 2 Engineering Research Center of Bone and Joint Precision Medicine, Ministry of Education, Beijing, China

**Keywords:** femoral neck fracture, comminuted posteromedial cortex, femoral neck system, cannulated screw, finite element analysis

## Abstract

**Background:**

The purpose of this study was to explore the biomechanical property and clinical efficacy of femoral neck system (FNS) with an additional cannulated screws (CS) in the treatment of unstable femoral neck fracture (FNFs) with comminuted posteromedial cortex.

**Methods:**

Firstly, we developed a model of Pauwels type III FNF with comminuted posteromedial cortex for the finite element analysis (FEA). Two experimental models were set up: the FNS model and the FNS + CS model. The von Mises stress on the proximal femur, implant and the total displacement of the device components were evaluated for both FNS and FNS + CS models. Secondly, we retrospectively included the cases of vertical FNFs with comminuted posteromedial cortex by FNS or FNS + CS fixation in our hospital from January 2020 to December 2023. In this study, demographic information, femoral neck shortening, Harris score of hip joint function, and postoperative complications were collected and compared.

**Results:**

The FEA results showed similar peak von Mises stress of the implant in two models and the additional CS could share the stress concentration with the FNS in the FNS + CS model. In terms of proximal femur, the maximum von Mises stress of the FNS model increased by 15.43% when compared with the FNS + CS model, and the magnitude of these two models were 83.02 MPa and 71.92 MPa, respectively. Furthermore, the maximum displacement in the FNS + CS model was much smaller than that in the FNS model. Clinically, the femoral neck shortening distance was significantly longer in the FNS group (5.62 ± 3.32 mm) than that in the FNS + CS group (3.49 ± 2.01 mm) (*p* = 0.027). Furthermore, the incidence of moderate to severe shortening (≥5 mm) was significantly higher in the FNS group compared with the FNS + CS group (*p* = 0.039). Moreover, the patients in the FNS + CS group had a higher Harris score than patients in the FNS group (91.97 vs. 88.56, *p* = 0.003).

**Conclusion:**

Compared to the FNS alone, the FEA results showed that the FNS + CS had better biomechanical properties and the clinical results showed that the FNS + CS had a shorter femoral neck shortening and higher Harris score in treating unstable FNFs with comminuted posteromedial cortex.

## Introduction

Femoral neck fractures (FNFs) are very common in the elderly patients, which are usually caused by low-energy injury. Whereas, FNFs in young and middle-aged adults are uncommon, which often result from high-energy injury (Roser et al., 2024). As a result, the high-energy injury can easily lead to severe bone fracture of the femoral neck. Given the high functional demands after surgery and the limited lifespan of artificial joints, surgical internal fixation remains the primary treatment choice for young and middle-aged patients with femoral neck fractures (FNFs) (Chan, 2019). At present, there are still many problems in the internal fixation for these unstable FNFs, and the incidence of postoperative complications is notably high (Yang et al., 2013). Therefore, it is very important to choose a proper implant for unstable FNFs to decrease the incidence of postoperative complications.

The femoral neck system (FNS), integrating the advantages of minimally invasive cannulated screws (CS) and the stability of dynamic hip screws (DHS), is a newly designed implant for FNFs which has the advantages of superior resistance to rotation and shear forces (Stoffel et al., 2017; Huang et al., 2023). Nevertheless, FNS-managed FNF patients continued to exhibit a relatively high incidence of postoperative complications (Davidson et al., 2022; Schuetze et al., 2023). Several studies emphasize the significance of posterior comminution in femoral neck fractures, considering it as a cause of unstable fixation (Rawall et al., 2012). The unstable FNFs can become even more unstable due to the loss of complete posterior cortical support for external rotation. In clinical practice, we find that the FNS fixation could provide only single-plane stability, while FNFs with posterior medial cortical defects are usually a three-dimensional spatial configuration. Single plane of the FNS cannot provide complete and effective mechanical support. To solve this problem, the additional CS has been introduced into clinical practice (Lin et al., 2024). By providing additional fixation points, the additional CS enhances the anti-shortening force, and the mechanical plane of the CS is not in the same plane with the FNS, forming angle stable fixation, which may effectively reduce postoperative complications.

Currently, it is still unclear whether an additional CS is necessary for unstable FNFs with comminuted posteromedial cortex by FNS fixation. This study aims to achieve the following objectives by constructing an unstable FNF with comminuted posteromedial cortex model and collecting clinical data of these patients: 1. Finite element analysis method is used to analyze and compare the biomechanical stability of FNS and FNS with an additional CS in the treatment of FNF with comminuted posteromedial cortex. 2. The clinical efficacy of FNS and FNS with an additional CS is evaluated and compared in treating FNFs with comminuted posteromedial cortex in young and middle-aged adults.

## Materials and methods

All methods and experimental protocols in this study were performed in accordance with relevant guidelines and regulations, and approved by the Institutional Ethical Review Board of Peking University Third Hospital.

### Finite element analysis (FEA)

#### Establishment of the proximal femoral model and fracture model

In the current study, computed tomography images of a Sawbone femur (Model 3,406, fourth Generation Sawbone, Vashon, WA, United States) were acquired and imported into Mimics 19.0 (Materialise Group, Leuven, Belgium) to generate a three-dimensional model. This 3D finite element model was subsequently utilized in a prior investigation (Zhou et al., 2025).

**FIGURE 1:**
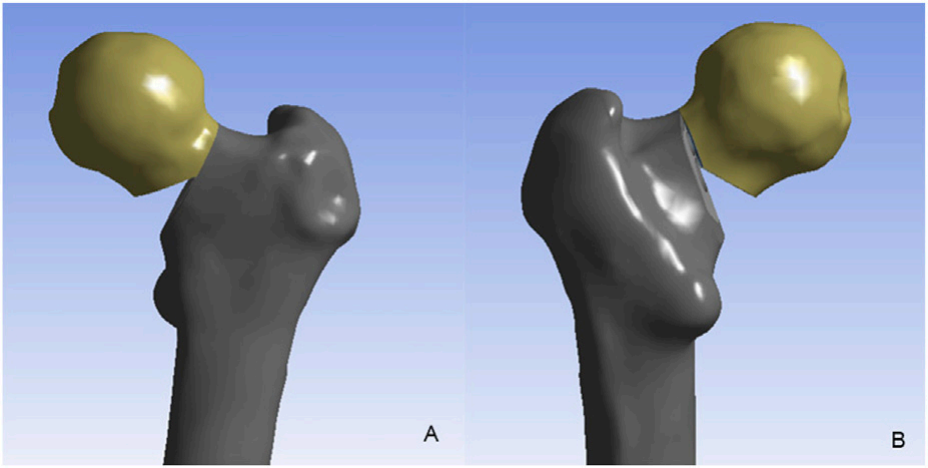
The unstable femoral neck fracture with comminuted posteromedial cortex. **(A)** the anterior view; **(B)** the posterior view.

Surface imperfections (including spikes and intersections) in the 3D model of the proximal femur were rectified using Geomagic Studio 12.0 (Raindrop Inc., United States). Following surface refinement, a smoothed solid model was generated and imported into SolidWorks (Dassault Systèmes SolidWorks Corp., United States). To simulate femoral neck fracture (FNF) models with comminuted posteromedial cortex: 1. A primary 20° osteotomy was performed at the femoral neck center relative to the shaft axis, establishing a Pauwels type III fracture pattern; 2. Posteromedial cortical comminution was then created by resecting two wedges: a 30° distal wedge and a 15° posterior wedge (Relative to the initial osteotomy plane, following Windolf’s protocol) (Windolf et al., 2009). (Figure 1)

**FIGURE 2:**
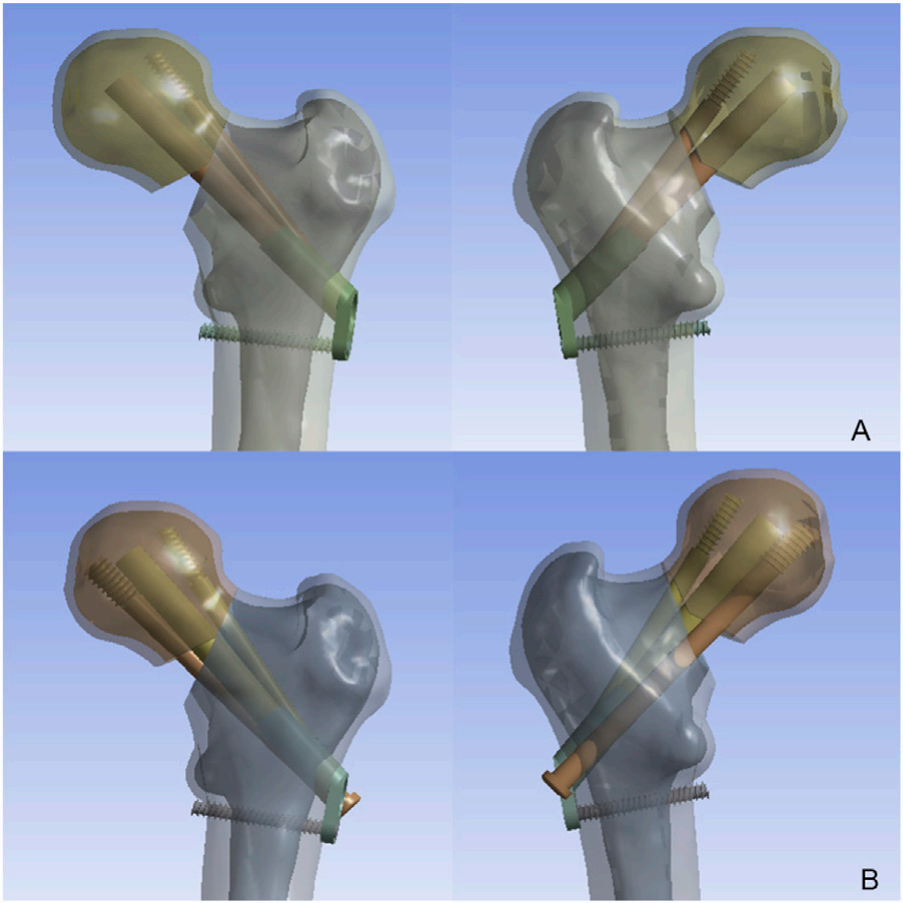
The model of femoral neck fracture with comminuted posteromedial cortex implanted with the FNS and CS. **(A)** The FNS model; **(B)** The FNS + CS model.

According to the DePuy Synthes (West Chester, PA, United States) CS parameters, the screws featured a 7.3 mm threaded diameter, 16 mm length, and 4.8 mm unthreaded diameter. In the FNS model, a 10-mm lag screw was positioned at 130° to the locking plate, accompanied by a 6.4-mm anti-rotation screw angled at 7.5° relative to the lag screw. At the distal end, one hole was made for a 5-mm locking screw. Then, the CS and FNS were virtually inserted into the FNF fracture model, and the FNS model and FNS with an additional CS (FNS + CS) model were created (Figure 2). The models were then transferred to ANSYS Workbench 14.5 (ANSYS Inc., Canonsburg, PA) for simulation.

In the present study, all materials were assumed to be homogeneous, isotropic, and linearly elastic (Henschel et al., 2016). The material properties of the femur and implant materials were summarized in Table 1 (Kwak et al., 2018). Based on established contact setup methods from previous studies, binding contact was applied between the internal fixation screw and the femur, while friction contact with a coefficient of 0.46 was used on the fracture surface (Zhou et al., 2020; Sensoz et al., 2018).

**TABLE 1:** Material properties used in the simulations in this study.

Material	Young’s modulus (Mpa)	Poisson’s ratio
Cortical bone	17,000	0.33
Cancellous bone	1,000	0.3
FNS/CS (Ti-6Al-7NB)	110,000	0.35

#### Boundary and loading conditions

For boundary conditions, the distal end of the femur was fully constrained. The applied loading forces on the femur presented the loads during one-legged stance (Lotz et al., 1995). A joint reaction force of 1260N ({x, y, z} = {365.4, −126, −1,197}) was applied at the femoral head (1.8 times body weight) (Lee et al., 2016).

#### Observation index

In the finite element analysis, the peak von Mises stress on the proximal femur and implant, the total displacements of the models were selected as indices of the stability. They were evaluated and compared under the one-legged stance.

### Clinical research

#### Source of patients

This study was performed at a teaching hospital from January 2020 to December 2023. The inclusion criteria were as followed: (1) Age >18 years and <60 years; (2) Acute femoral neck fracture (<2 weeks from injury); (3) X-ray examination showed that the fracture was significantly displaced, Pauwels type III (Pauwells angle >50°); (4) CT examination showed comminuted posteromedial cortex of the FNF; (5) Prior to the fracture, the proximal anatomy of the femur was normal, and there was no history of hip disease; (6) The FNF was fixed by FNS or FNS with an additional CS; (7) A minimum follow-up period of 12 months or until the time of failure leading to revision surgery. Patients with delayed fractures, history of hip joint disease, pathological fractures, combined with ipsilateral lower limb fractures and incomplete follow-up data were excluded.

The collected clinical data included: patient’s gender, age, body mass index (BMI), fracture side, cause of injury, fracture classification based on the Garden and Pauwels systems, the surgical time, internal fixation method and blood loss during surgery.

#### Surgical procedure

All of the surgeries were performed by experienced orthopaedic surgeons. Spinal anesthesia or general anesthesia was used. Each FNF patient was placed on the traction table in the supine position. Reduction of the FNF was confirmed via intraoperative C-arm fluoroscopy, and the quality of fracture reduction was assessed by determining the Garden index. The Kirschner wire was temporarily inserted for fixation. Routine surgical procedures, including guide pin insertion, depth measurement, drilling, and installation of the FNS device, were performed for implantation of the FNS according to the manufacturer’s protocol. For the FNS + CS group, additional steps of guide pin insertion, depth measurement, and drilling were performed, followed by implantation of the additional CS. Before the wound was closed, the reduction and proper insertion of FNS components were confirmed via C-arm fluoroscopy. Postoperatively, patients received antibiotic prophylaxis and deep vein thrombosis (DVT) prophylaxis, with partial weight-bearing initiated after evidence of fracture healing was observed on radiographs and total weight-bearing commenced once clinical fracture healing was confirmed. Follow-up evaluations were conducted at 1, 3, 6, and 12 months postoperatively and annually thereafter.

### Outcomes

#### Reduction quality assessment

Garden index This commonly used classification system categorized FNFs into four levels based on the extent of displacement and the quality of reduction (GARDEN, 1964). Employed to predict fracture healing difficulty and potential complications, this method relied on routine postoperative radiographs to assess reduction quality. The grading system was: Grade I (AP 160°, Lateral 180°); Grade II (AP 155°, Lateral 180°); Grade III (AP < 155° or Lateral > 180°); Grade IV (AP < 150°, Lateral > 180°). Grades III and IV signified poor reduction quality.

#### Tip apex distance (TAD)

Immediately after surgery, anteroposterior (AP) and lateral radiographs of the hip joint were obtained. On these views, the distance from the tip of the screw to the apex of the intersection, formed by the femoral head-neck axis and the articular surface of the femoral head, was measured. Specifically, the anteroposterior distance (Xap) and lateral distance (Xlat) were recorded. The actual width of the screw (Dtrue) was used to correct the magnification factor of the screw width measured on the AP (Dap) and lateral (Dlat) views. The sum of these corrected distances was then calculated to determine the TAD value, using the formula: TAD = (Xap × Dap)/Dtrue + (Xlat × Dlat)/Dtrue (Subasi et al., 2022).

#### Femoral neck shortening

The amount of femoral neck shortening was measured on radiographs on anteroposterior (AP) radiographs using the method described by Zlowodzki et al. (Zlowodzki et al., 2008). The healthy side was served as a reference for measuring horizontal (X-axis) and vertical (Y-axis) femoral head shortening on the affected side. Femoral neck axial shortening (Z-axis) was derived from formula Z = Ysin(θ) + Xcos(θ), where θ is the angle between the Y-axis and the femoral neck axis. Shortening severity, defined by the Z value, was classed as mild (<5 mm), moderate (5–10 mm), or severe (>10 mm).

#### Postoperative complications

This included implant failure (e.g., fracture or deformation), delayed or nonunion, implant displacement relative to the bone (without femoral head penetration), deep incisional infection, implant penetration into the joint cavity via the femoral head, avascular necrosis of the femoral head, and the requirement for secondary total hip arthroplasty (Lin et al., 2025).

### Statistical analysis

Continuous data were presented as mean ± standard deviation (SD) for normally distributed variables or median with interquartile range (IQR) for non-normally distributed variables. The normality test was assessed using the Shapiro-Wilk test. Student’s t-test was applied to normally distributed data, while nonparametric methods were used for non-normally distributed data. Categorical variables were analyzed with the chi-square test, and ranked data were evaluated using the Mann-Whitney U test. A significance level of *p* < 0.05 was considered statistically significant. Statistical analyses were performed with IBM SPSS Statistics (Version 26.0).

## Results

### FEA results

**FIGURE 3:**
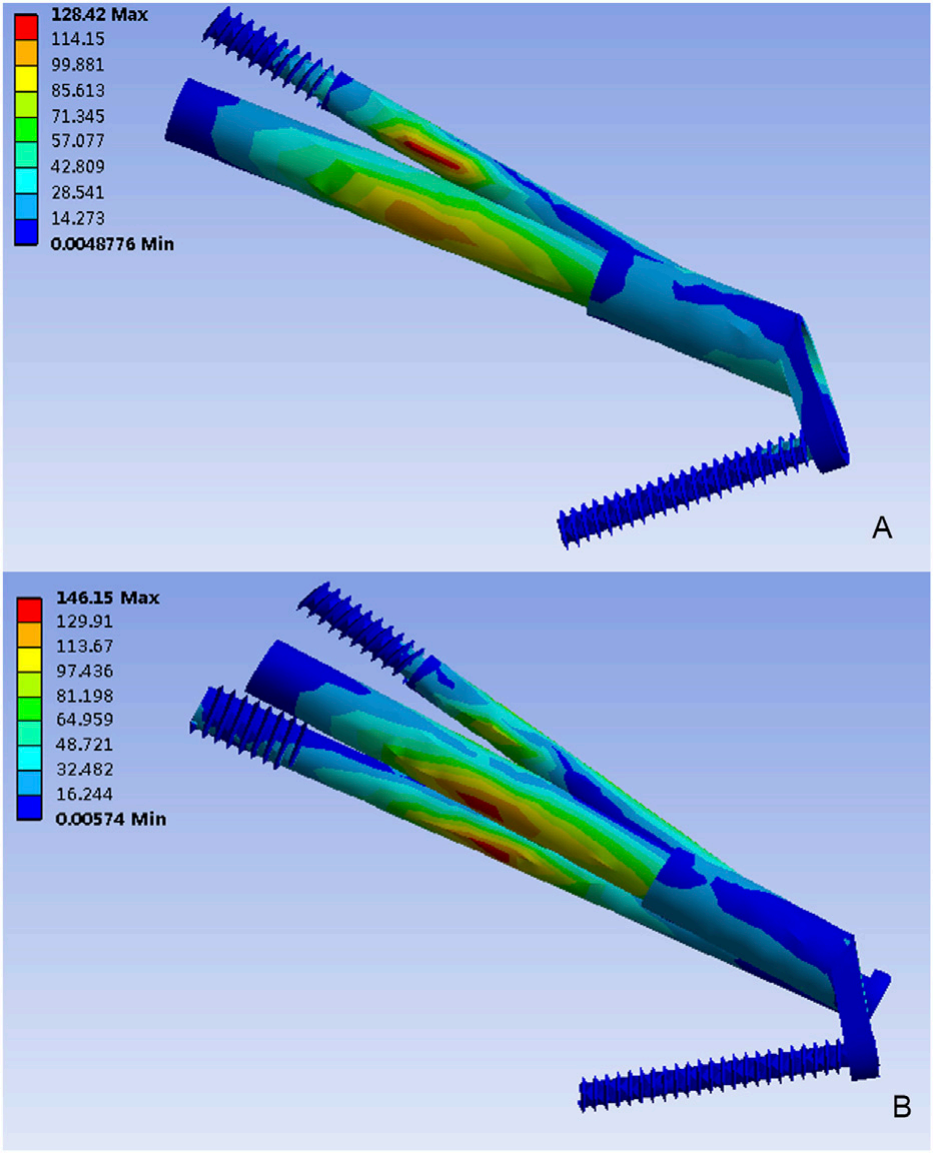
The Von Mises stress distribution (MPa) on the implant. **(A)** The FNS model; **(B)** The FNS + CS model.

The von Mises stress distributions for two internal fixation models were assessed and shown in Figure 3. In the FNS model, the stress concentration area was located at the anti-rotational screw and the peak von Mises stress was 128.42 MPa. In the FNS + CS model, the peak von Mises stress was 146.15 MPa, whereas the stress concentration area was located at the CS and sliding hip screw. Therefore, the CS could share the stress concentration with the FNS.

**FIGURE 4:**
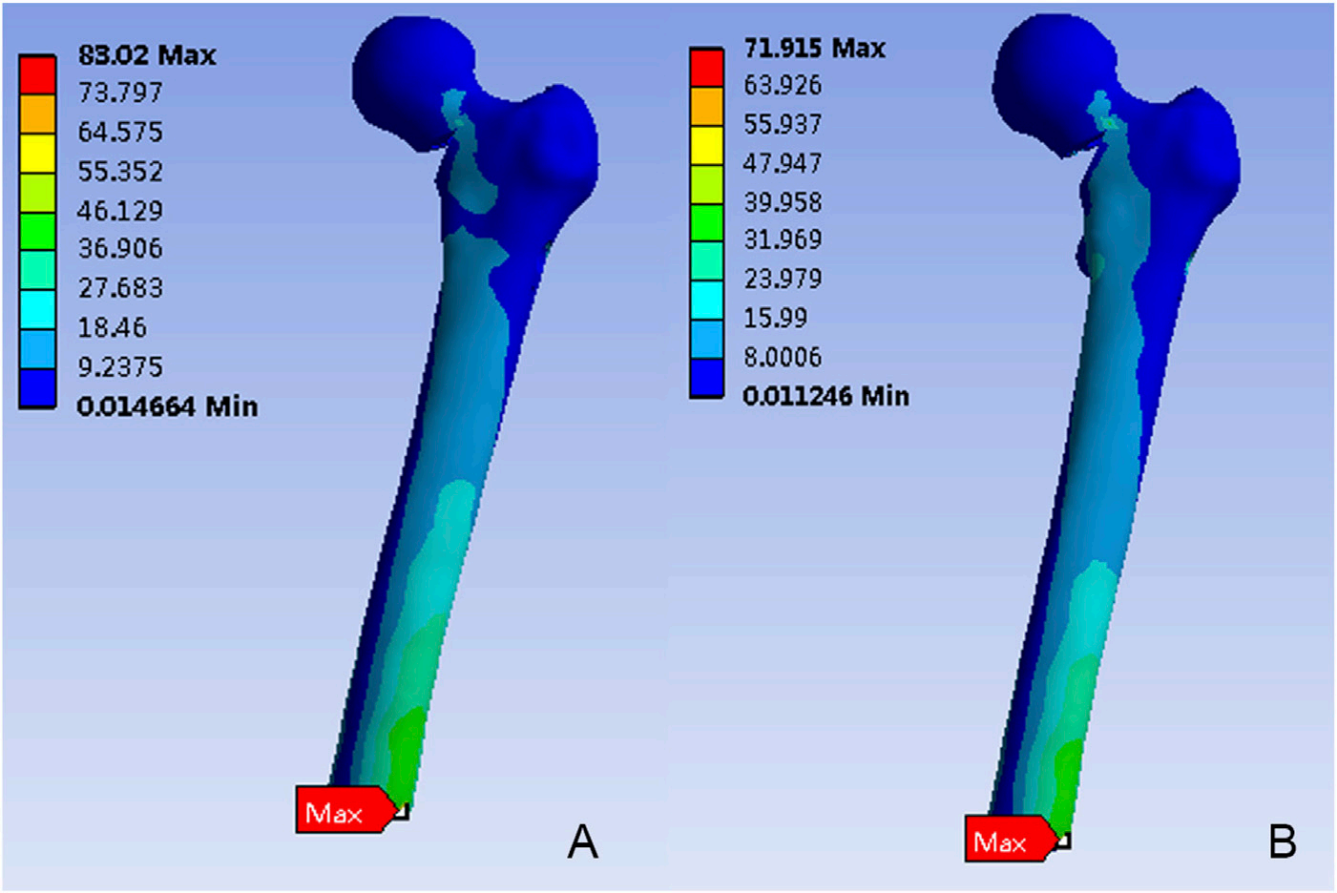
The Von Mises stress distribution (MPa) on the proximal femur. **(A)** The FNS model; **(B)** The FNS + CS model.

Figure 4 depicted the von Mises stress distribution in the proximal femur for FNS and FNS + CS. While the stress concentration area was located at the inferior femur in both models, differences in the distribution were observed. The maximum von Mises stress in the FNS model was 83.02 MPa, which represented a 15.43% increase compared to the FNS + CS model (71.92 MPa).

**FIGURE 5:**
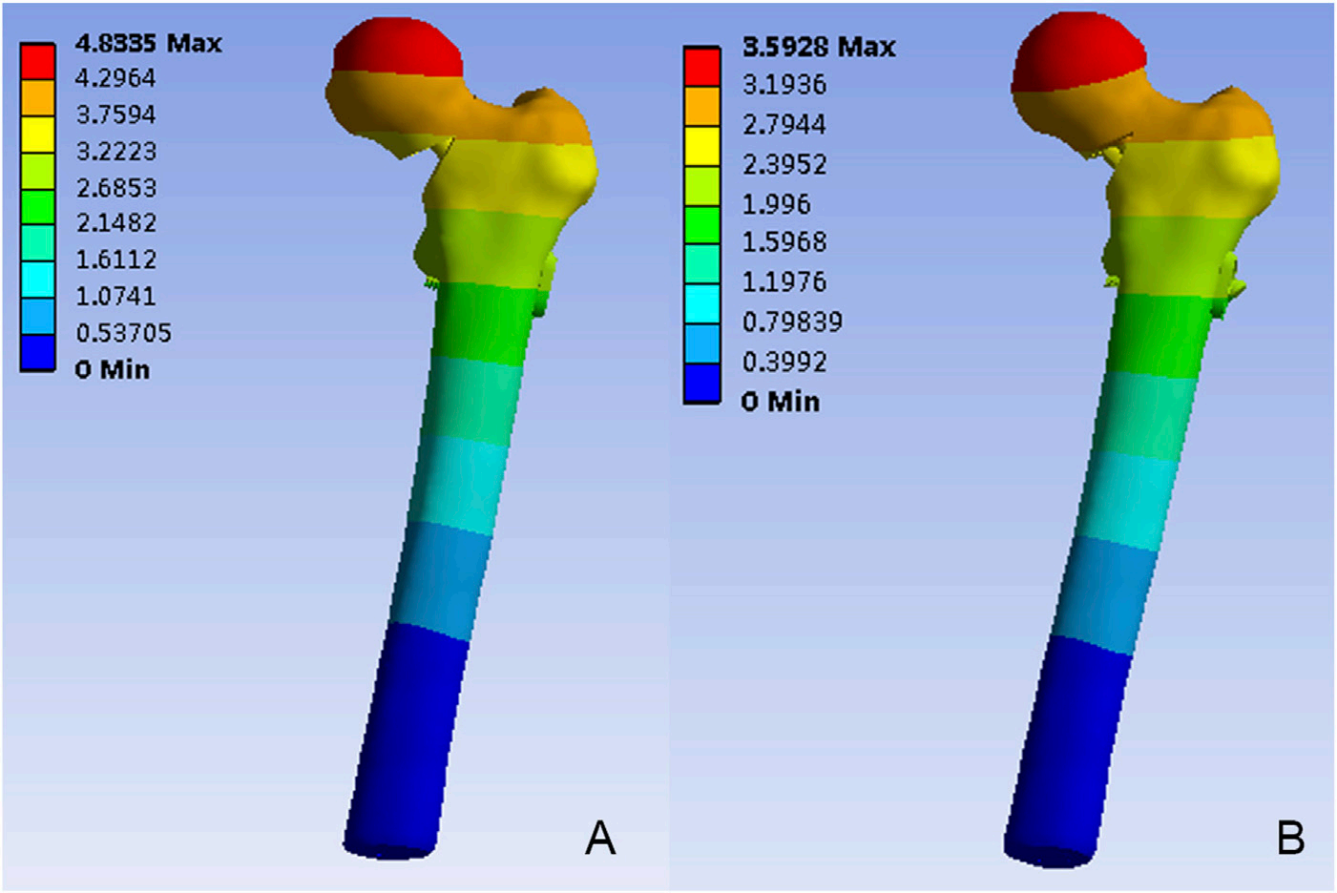
Displacement distribution (mm) in both models. **(A)** The FNS model; **(B)** The FNS + CS model.

Figure 5 depicted the model displacement distribution in two models. For both models, the maximum displacements occurred at the top of the femoral head. The maximum model displacement of the FNS model increased by 25.67% when compared with the FNS + CS model, and the magnitude of these two models were 4.83 mm and 3.59mm, respectively.

### Clinical results

A total of 41 patients were included in this study, with 25 patients in the FNS group and 16 patients in the FNS + CS group. (Table 2). Clinical characteristics of patients in two groups were summarized in Table 1. Patients in the FNS + CS group were slightly older than those in the FNS group (49.38 vs. 48.44, *p* = 0.816). Of these patients, 7 (28%) were female patients in the FNS group, and 4 (25%) were female patients in the FNS + CS group. The average BMI was 23.85 (±2.49) kg/m2 and 25.32 (±3.77) kg/m2 in the FNS group and FNS + CS group, respectively (P = 0.140). With respect to the injury side and mechanism of injury, 18 (72%) had left side injury and 17 (68%) suffered from fall from height injury in the FNS group, and 9 (56.3%) had left side injury and 13 (81.3%) suffered from fall from height injury in the FNS + CS group. Patients belonging to the FNS group had a similar level of surgical time (60.64 vs. 63.06, *p* = 0.744), intraoperative bleeding (34.00 vs. 33.13, *p* = 0.892), TAD (16.71 vs. 16.36, *p* = 0.831), healing time (8.08 vs. 8.38, *p* = 0.071) and length of stay (3.32 vs. 3.63, *p* = 0.401) compared with those in the FNS + CS group. In addition, similar levels of Garden classification and reduction quality were found in both the FNS group and FNS + CS group (*p* > 0.05).

**TABLE 2:** Comparison of the clinical characteristics between the FNS group and FNS + CS group.

Characteristic	FNS group (N = 25)	FNS + CS group (N = 16)	P Value
Age (mean years±SD)	48.44 ± 14.09	49.38 ± 9.21	0.816
Women, N (%)	7 (28.0)	4 (25.0)	0.833
BMI (kg/m2)	23.85 ± 2.49	25.32 ± 3.77	0.140
Injury side (left/right)			0.300
Left, N (%)	18 (72.0)	9 (56.3)	
Right, N (%)	7 (28.0)	7 (43.8)	
Mechanism of injury			0.350
Fall from height, N (%)	17 (68.0)	13 (81.3)	
Motor vehicle collision, N (%)	8 (32.0)	3 (18.8)	
Smoking, N (%)	2 (8.0)	3 (18.8)	0.305
Drinking, N (%)	2 (8.0)	2 (12.5)	0.636
Garden classification			0.444
Garden III, N (%)	14 (56.0)	7 (43.8)	
Garden IV, N (%)	11 (44.0)	9 (56.3)	
Surgical time (mm)	60.64 ± 25.59	63.06 ± 18.02	0.744
Intraoperative bleeding (mm)	34.00 ± 23.63	33.13 ± 12,50	0.892
Reduction quality			0.395
Neutral support, N (%)	16 (64.0)	8 (50.0)	
Positive support, N (%)	6 (24.0)	7 (43.8)	
Negative support, N (%)	3 (12.0)	1 (6.3)	
TAD (mm)	16.71 ± 5.57	16.36 ± 4.19	0.831
Healing time (week)	8.08 ± 0.28	8.38 ± 0.72	0.071
Length of stay (day)	3.32 ± 1.28	3.63 ± 0.81	0.401
Femoral neck shortening (mm)	5.62 ± 3.32	3.49 ± 2.01	0.027*
Femoral neck shortening grade			0.039*
Mild (<5 mm), N (%)	11 (44.0)	13 (81.3)	
Moderate (5–10 mm), N (%)	9 (36.0)	3 (18.8)	
Severe (>10 mm), N (%)	5 (20.0)	0 (0)	
Harris score	88.56 ± 2.87	91.97 ± 4.06	0.003*
Postoperative complications	2 (8.0%)	2 (12.5%)	0.636

Abbreviations: BMI, body mass index; FNS, femoral neck system; CS, cannulated screw; TAD, tip apex distance; SD, standard deviation.

*The difference was significant.

**FIGURE 6:**
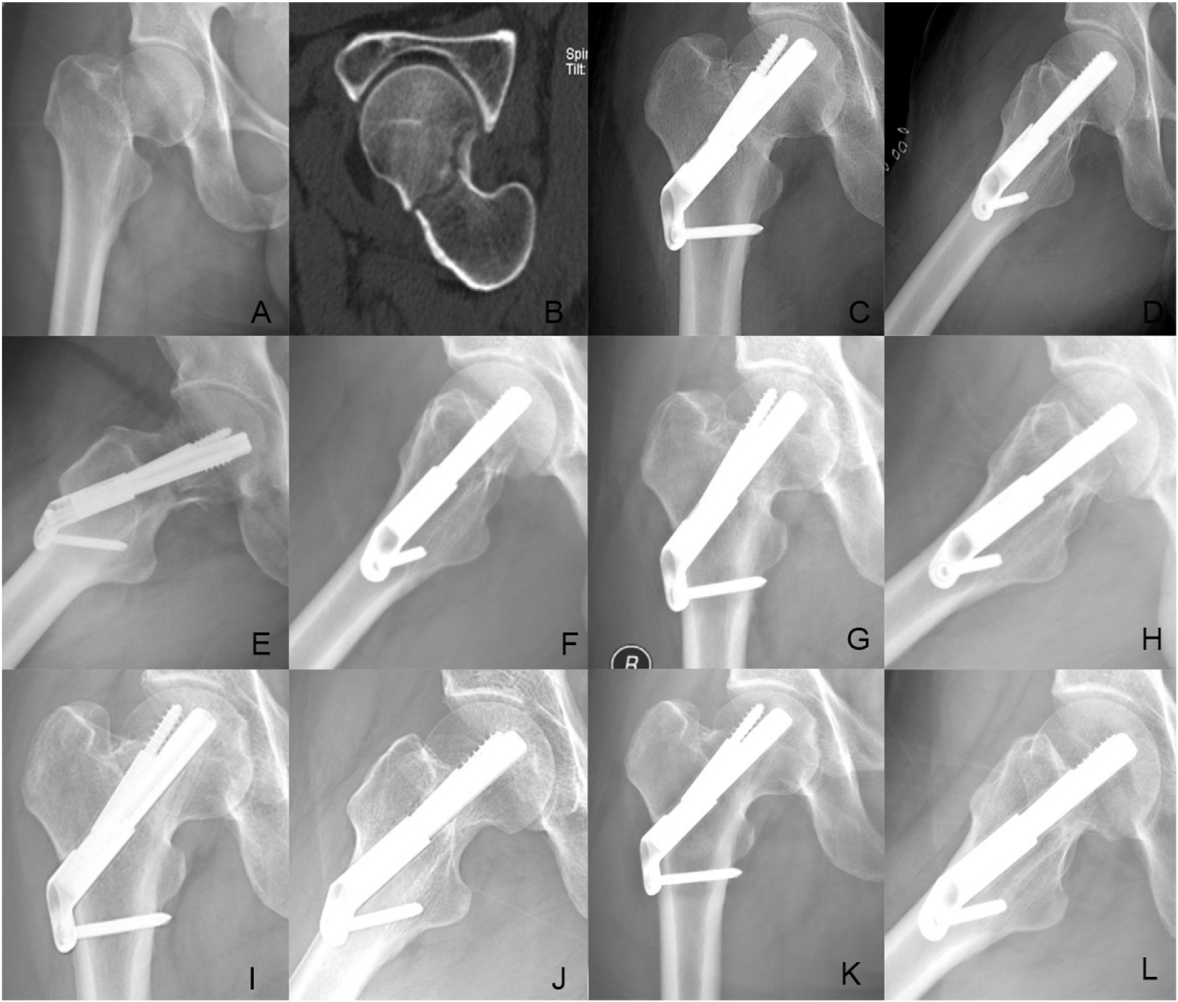
Case presentation: a 42 years old man with right femoral neck fracture fixed with FNS. **(A,B)** Preoperative anteroposterior radiograph and CT scan showed the right FNF with posteromedial cortex fracture. **(C,D)** The anteroposterior and lateral X-ray with FNS 1 day after surgery; **(E,F)** The anteroposterior and lateral X-rays 1Dmonth after surgery; **(G,H)** The anteroposterior and lateral X-rays 3 months after surgery; **(I,J)** The anteroposterior and lateral X-rays 6 months after surgery; **(K,L)** The anteroposterior and lateral X-rays 12 months after surgery.

**FIGURE 7:**
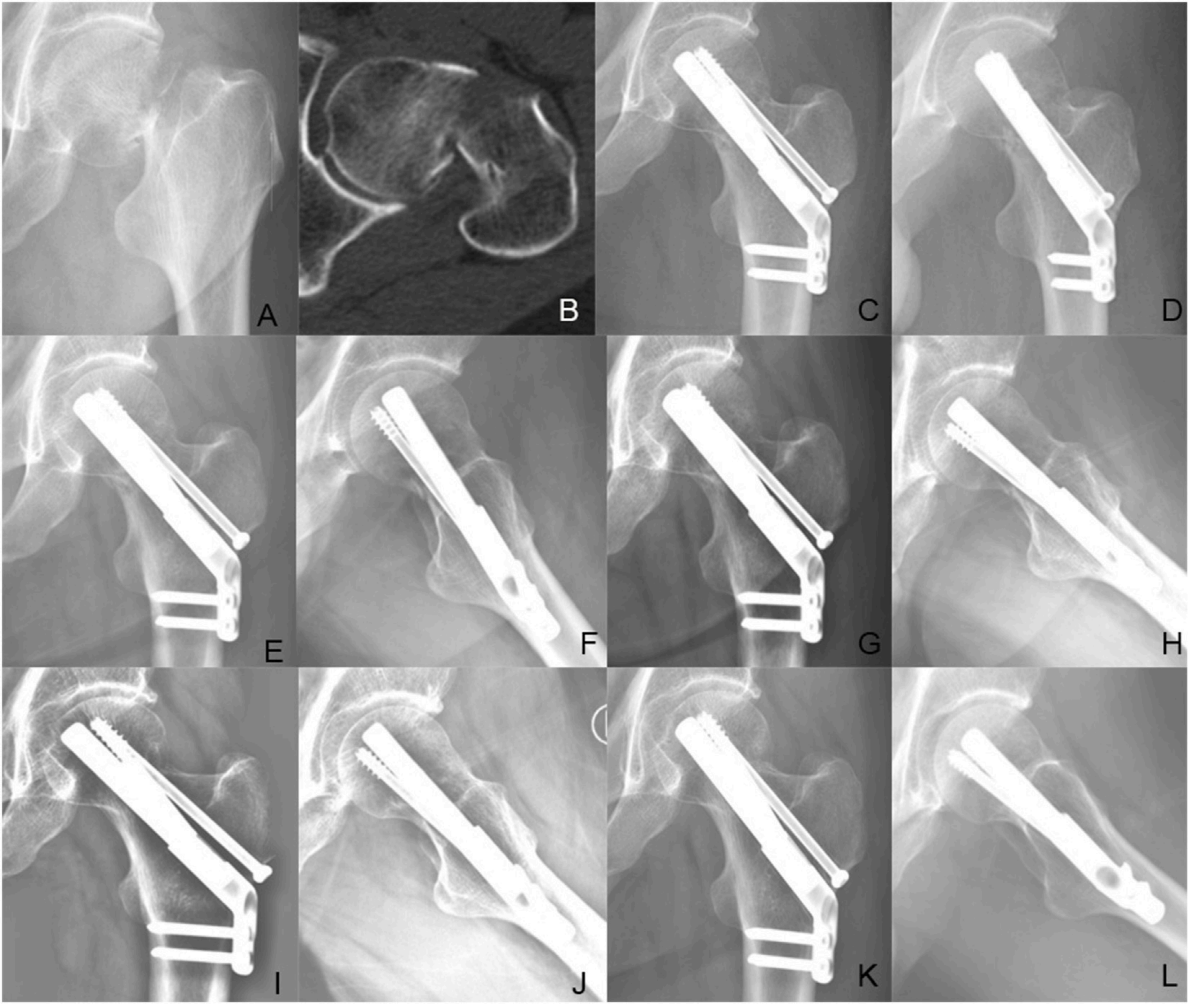
Case presentation: a 56 years old man with left femoral neck fracture fixed with FNS + CS. **(A,B)** Preoperative anteroposterior radiograph and CT scan showed the right FNF with posteromedial cortex fracture. **(C,D)** The anteroposterior and lateral X-ray with FNS 1 day after surgery; **(E,F)** The anteroposterior and lateral X-rays 1 month after surgery; **(G,H)** The anteroposterior and lateral X-rays 3 months after surgery; **(I,J)** The anteroposterior and lateral X-rays 6 months after surgery; **(K,L)** The anteroposterior and lateral X-rays 12 months after surgery.

**FIGURE 8:**
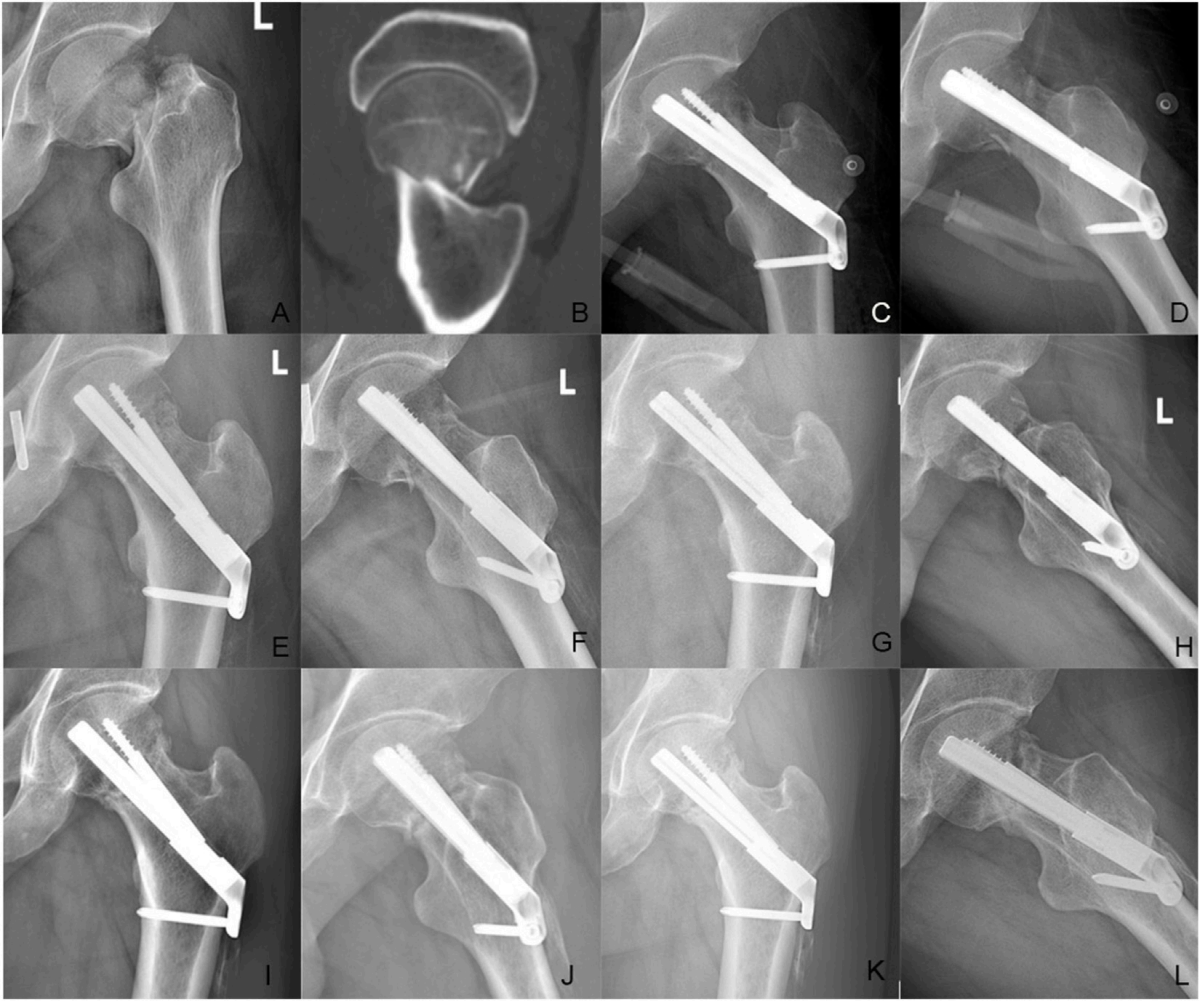
Case presentation: a 35 years old man with left femoral neck fracture fixed with FNS. **(A,B)** Preoperative anteroposterior radiograph and CT scan showed the left FNF with posteromedial cortex fracture. **(C,D)** The anteroposterior and lateral X-ray with FNS 1 day after surgery; **(E,F)** The anteroposterior and lateral X-rays 1 month after surgery; **(G,H)** The anteroposterior and lateral X-rays 3 months after surgery; **(I,J)** The anteroposterior and lateral X-rays 6 months after surgery; **(K,L)** The anteroposterior and lateral X-rays 12 months after surgery.

**FIGURE 9:**
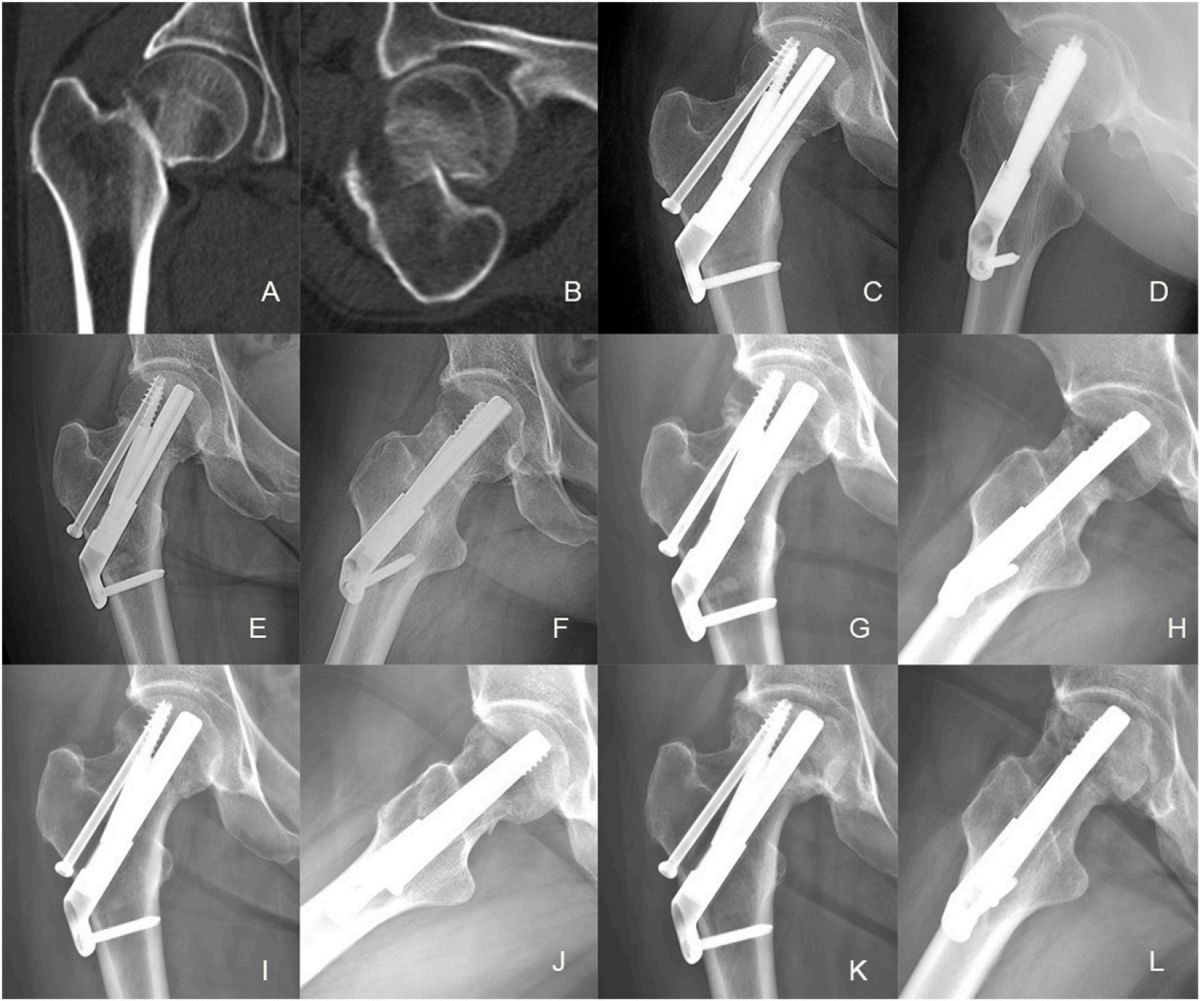
Case presentation: a 46 years old woman with right femoral neck fracture fixed with FNS. **(A,B)** Preoperative CT scan showed the right FNF with posteromedial cortex fracture. **(C,D)** The anteroposterior and lateral X-ray with FNS 1 day after surgery; **(E,F)** The anteroposterior and lateral X-rays 1 month after surgery; **(G,H)** The anteroposterior and lateral X-rays 3 months after surgery; **(I,J)** The anteroposterior and lateral X-rays 6 months after surgery; **(K,L)** The anteroposterior and lateral X-rays 12 months after surgery.

In addition, the femoral neck shortening distance was significantly longer in the FNS group (5.62 ± 3.32 mm) than that in the FNS + CS group (3.49 ± 2.01 mm) (*p* = 0.027). Furthermore, the incidence of moderate to severe shortening (≥5 mm) was significantly higher in the FNS group compared with the FNS + CS group (*p* = 0.039). These findings suggested that the additional CS might be more effective in reducing femoral neck shortening compared with the FNS along. Moreover, the patients in the FNS + CS group had a higher Harris score than patients in the FNS group (91.97 vs. 88.56, *p* = 0.003). In terms of postoperative complications, the one-year follow-up results showed that one case of fracture nonunion occurred in the FNS group and the avascular necrosis of the femoral head occurred in one case in the FNS + CS group. In the two-year follow-up results showed that one case of fracture nonunion and one case of femoral head avascular necrosis occurred in the FNS group, and two cases of the avascular necrosis of the femoral head occurred in the FNS + CS group. Typical successful cases were shown in Figures 6, 7. The typical unsuccessful cases and pictures of two groups were shown in Figures 8, 9.

## Discussion

The substantial energy trauma of the fracture itself and the inappropriate internal fixation system are related to the functional recovery of femoral neck fractures (Jiang et al., 2023; Robinson et al., 1995). The high energy trauma might result in more displaced and comminuted fracture. Compared to non-displaced fracture, the displaced FNF (Garden type III and Ⅳ) has a higher risk of fracture nonunion and femoral head necrosis (Xin et al., 2023). For displaced FNF, the external rotational stress could lead to anterior femoral cortex fracture and the posterior cortex comminution. Previous study reported that up to 70% of unstable displaced FNSs had posterior cortex comminution or bone defects (Liu et al., 2019). Anatomical reduction and stable fixation for these extremely unstable FNFs with posterior bone defect was vital for fracture union and satisfactory function recovery. In this study, the FEA results showed that the additional CS could shear the stress conduction and decrease the total model displacement. The clinical results showed that patients in the FNS + CS group had a lower risk of femoral neck shortening and higher Harris score.

Currently, it was still unclear which implant was the best choice for these extremely unstable FNFs. These fractures posed great challenges for surgical reduction and fixation for their inherent instability, resulting in great difficulty in consistently achieving a stable construct and maintaining reduction. Inadequate stabilization could result in displacement, disruption of blood supply, and fixation failure, with subsequent development of nonunion and avascular necrosis (AVN) of the femoral head. Theoretically, the ideal implant should maintain angular and longitudinal stability, resist forces traversing the fracture site, and permit controlled micro-dynamization (Lazaro et al., 2016). Currently, multiple cannulated cancellous screws (MCS) were the predominant fixation method for femoral neck fractures. However, this construct lacked inherent stability between screws, and screw placement was highly dependent on surgeon technique and anatomical variables. Consequently, MCS demonstrated poor resistance to vertical shear and torsional forces, potentially leading to screw loosening, fracture displacement, femoral head avascular necrosis, nonunion, and femoral neck shortening. Previous studies had reported that the MCS was unable to firmly fix Pauwels type III FNF and showed more postoperative complications (Samsami et al., 2015; Ma et al., 2018). The DHS-blade, offering superior stability, was established as the European gold standard for treating unstable femoral neck fractures. However, it required a larger skin incision and more extensive soft tissue dissection (Bhan et al., 2009). Integrating the benefits of minimally invasive surgery and angle-stable fixation, the FNS utilized a unique design: an anti-rotational screw locked into the bolt, allowing both components to slide dynamically within the plate barrel. This dynamic fixation, combined with a fixed plate and integrated lag screw, significantly strengthened angular stability. This effectively prevented reduction loss and provided superior anti-rotation. Clinical evidence established FNS as superior to MCS fixation for femoral neck fractures, associated with faster patient recovery and lower complication rates (Huang et al., 2023; Xu et al., 2024). However, postoperative complications could occur in Pauwels type III FNF after FNS fixation, especially for unstable FNF with comminuted posteromedial cortex (Guo et al., 2024). A few researchers recommended an additional CS to increase the fracture fixation stability (Su et al., 2023). This might be the additional CS could achieve different plane fixations and increase angle stability.

For complex femoral neck fractures, postoperative biomechanical stability directly correlated with positive treatment outcomes. Biomechanically, the additional CS of the Femoral Neck System (FNS) enhanced screw-screw spacing, mitigated stress concentration, and transformed shear forces into compressive forces. This stabilized the fracture site, created an optimal mechanical environment for bone healing, and reduced postoperative fixation failure risks. To evaluate the stability of unstable FNFs featuring comminuted posteromedial cortex and stabilized with FNS or FNS + CS, the primary indicator was the total displacement of the proximal femur and the fixation device under load. In this study, the FEA results showed that the additional CS could share the stress concentration with the FNS. Furthermore, the maximum model displacement of the FNS model increased by 25.67% when compared with the FNS + CS model, showing good postoperative stability. For unstable FNFs, the lack of posterior support could lead to an imbalance in force loading on the femoral head, which might result in varus or cutout complications. In the case of these complex femoral neck fractures, the implant served as the sole supportive mechanism capable of providing effective support. Additionally, unstable FNFs could generate shear forces at the fracture site, causing medialization and shortening of the femoral shaft, accompanied by varus angulation and external rotation of the proximal fragment (Gao et al., 2025). Therefore, minimizing interfragmentary movement was essential for achieving relative stability during early fracture healing. In this study, the FNS augmented with additional CS (FNS + CS) effectively stabilized the femoral head fragment, demonstrating significantly reduced fracture displacement. These findings suggested that FNS + CS might serve as a viable alternative for unstable femoral neck fractures with posterior bone defects due to its superior biomechanical stability.

Femoral neck shortening remained a common surgical complication of femoral neck fractures, which adversely affected patient prognosis (Lee et al., 2024; Felton et al., 2019). The FNS featured a 20 mm sliding compression space which could prevent excessive sliding and minimize femoral neck shortening, and its dynamic compression mechanism at the fracture site might enhance fracture healing (Zhou et al., 2021). In this study, the FNS + CS group had significantly lower degrees of femoral neck shortening than the FNS group (*p* = 0.027). Furthermore, the incidence of moderate to severe shortening (≥5 mm) was significantly higher in the FNS group compared with the FNS + CS group (*p* = 0.039). These findings suggested that the additional CS might be more effective in reducing femoral neck shortening compared with the FNS along. Moreover, the patients in the FNS + CS group had a higher Harris score than patients in the FNS group (91.97 vs. 88.56, *p* = 0.003). The larger femoral neck shortening, especially shortening greater than 10 mm, could impact limb function. The FNS group had a higher incidence of severe femoral neck shortening than the FNS + CS group. However, the Harris score differences were small (∼3 points). The Harris score would be categorized as excellent (90–100), good (80–89), fair (70–79), or poor (<70). All patients in the FNS group and FNS + CS group had a good Harris score, although a statistical difference was found. The clinical meaningfulness of this improvement should be evaluated carefully by the orthorpeadic surgeons. The superior fixation stability achieved by FNS + CS likely contributed to enhance fracture healing and improve functional outcomes. However, these results should be interpreted with caution due to the smaller sample size in the FNS + CS group relative to the FNS group, warranting larger-scale studies to validate these observations. Furthermore, the additional CS had a risk of joint penetration, or weakening of bone stock for future arthroplasty. For non-elderly patients, a good reduction quality and a firm fixation method was the first choice (Roser et al., 2024). For these patients, a relatively good bone mineral density might decrease the risk of joint penetration. The orthopeadic surgeon should balance the benefit of better stability and potential risk of the additional CS.

The development of femoral head avascular necrosis after FNFs was associated with several risk factors, including initial fracture displacement, the quality of intraoperative reduction, internal fixation stability, and postoperative weight-bearing duration. Fracture could disrupt the blood supply to the femoral head. Although the bony structure might heal, restoring and reconstructing the blood supply remained challenging. Consequently, assessing femoral head necrosis necessitated a minimum follow-up period of 2 years (Parker et al., 2013). Previous studies had reported that the incidence of femoral head osteonecrosis ranging from 10% to 30% with any internal fixation method, and our results were comparable (Hoshino et al., 2016; Gardner et al., 2015). In present study, the incidence of femoral head osteonecrosis in the FNS + CS group was higher than that in the FNS group, yet no statistically significant difference was observed. Additional follow-up was also required to ascertain whether the femoral head osteonecrosis rate continued to increase in patients undergoing FNS and FNS + CS treatments. Most notably, surgeons should strive to achieve perfect reduction during surgery and use minimally invasive techniques to preserve the blood supply to the femoral head. Longer follow-up periods and large-sample prospective studies were required in the future.

This study has some limitations. First, the femur and implants were inherently anisotropic materials. Nevertheless, in this study, to reduce the complexity of analysis, they were simplified into uniform, isotropic, and elastic materials. Secondly, this study did not account for the influence of soft tissues, including muscles and skin surrounding the femur, on the forces experienced by the femur following internal fixation. The simplified model with isotropic and linear elastic materials and the omission of soft tissue constraints were significant limitations. Although this approach incorporated simplifications and introduced potential discrepancies with actual conditions, it still provided a distinct trend for the research focus. Thirdly, a notable limitation of this study was the absence of experimental validation to confirm the model’s accuracy. Nevertheless, since the study’s objective was to compare relative values under identical loading environments and boundary conditions, the omission of validation testing was deemed acceptable. Future studies with more physiologically accurate models and experimental validation (bench-top testing) were necessary to confirm these theoretical results. Fourth, the sample size was relatively limited and this was a retrospective study. The retrospective design might have introduced selection bias, as the internal fixation method was chosen based on clinical experience, with surgeons potentially opting for augmented fixation in more complex cases. To enhance the reliability of our findings, a randomized, multicenter prospective study was warranted. Fifth, the follow-up was short. A longer follow-up and large sample of prospective study was needed in the future. Sixth, during the measurement of the femoral neck shortening, neck shaft angle, and TAD, non-standard patient positioning during radiography might have influenced the measured values. However, to minimize this potential source of error, a single researcher conducted all measurements, and the results were averaged across three measurements for each patient.

## Conclusion

In summary, compared to the FNS alone, the FEA results showed that the additional CS could share the stress concentration with the FNS and exhibit smaller maximum model displacement in unstable femoral neck fractures with comminuted posteromedial cortex. This suggested that the additional CS might provide a better mechanical environment for fracture healing. Furthermore, the clinical results showed that the FNS in combination with an additional CS had a shorter femoral neck shortening and higher Harris score in treating FNFs with posteromedial defect compared with FNS alone. Therefore, the additional cannulated screw might be necessary for unstable FNFs with comminuted posteromedial cortex by femoral neck system (FNS) fixation.

## Data Availability

The raw data supporting the conclusions of this article will be made available by the authors, without undue reservation.

## Abbreviations

FNF, femoral neck fracture; FNS, femoral neck system; CS, cannulated screw; DHS, dynamic hip screw; FEA, finite element analysis; BMI, body mass index; TAD, Tip apex distance; SD, standard deviation.
